# Development and Verification of the Hypoxia- and Immune-Associated Prognostic Signature for Pancreatic Ductal Adenocarcinoma

**DOI:** 10.3389/fimmu.2021.728062

**Published:** 2021-10-06

**Authors:** Dongjie Chen, Hui Huang, Longjun Zang, Wenzhe Gao, Hongwei Zhu, Xiao Yu

**Affiliations:** Department of Hepatopancreatobiliary Surgery, The Third Xiangya Hospital, Central South University, Changsha, China

**Keywords:** pancreatic ductal adenocarcinoma, microenvironment, hypoxia, immune, prognosis

## Abstract

We aim to construct a hypoxia- and immune-associated risk score model to predict the prognosis of patients with pancreatic ductal adenocarcinoma (PDAC). By unsupervised consensus clustering algorithms, we generate two different hypoxia clusters. Then, we screened out 682 hypoxia-associated and 528 immune-associated PDAC differentially expressed genes (DEGs) of PDAC using Pearson correlation analysis based on the Cancer Genome Atlas (TCGA) and Genotype-Tissue Expression project (GTEx) dataset. Seven hypoxia and immune-associated signature genes (*S100A16*, *PPP3CA*, *SEMA3C*, *PLAU*, *IL18*, *GDF11*, and *NR0B1*) were identified to construct a risk score model using the Univariate Cox regression and the Least Absolute Shrinkage and Selection Operator (LASSO) Cox regression, which stratified patients into high- and low-risk groups and were further validated in the GEO and ICGC cohort. Patients in the low-risk group showed superior overall survival (OS) to their high-risk counterparts (*p* < 0.05). Moreover, it was suggested by multivariate Cox regression that our constructed hypoxia-associated and immune-associated prognosis signature might be used as the independent factor for prognosis prediction (*p* < 0.001). By CIBERSORT and ESTIMATE algorithms, we discovered that patients in high-risk groups had lower immune score, stromal score, and immune checkpoint expression such as PD-L1, and different immunocyte infiltration states compared with those low-risk patients. The mutation spectrum also differs between high- and low-risk groups. To sum up, our hypoxia- and immune-associated prognostic signature can be used as an approach to stratify the risk of PDAC.

## Introduction

Pancreatic ductal adenocarcinoma (PDAC) is such a devastating cancer that it accounts for the seventh biggest number of cancer deaths worldwide (1). Curative surgery remains the only potential cure for PDAC, but over 80% of them lose the opportunity with an advanced stage at the first diagnosis. Chemotherapy and radiotherapy for advanced PDAC patients have limited success due to the cancer microenvironment surrounding the tumor (2). Therefore, the prognosis of PDAC patients is extremely poor, with a 5-year overall survival (OS) rate of only 5% (3). Regarding the critical situation, the most urgent thing is to discover prognostic signature for PDAC patients, which will enable stratification of patients and precise treatment.

Hypoxia, as a major feature of cancerous microenvironment, exists in most malignancies, affecting carcinogenesis and developing tumorigenesis (4). The rapid proliferation of pancreatic tumors can easily cause oxygen stress and gradually form a hypoxic microenvironment (5). The hypoxic PDAC microenvironment has the following characteristics, including a median oxygen level of less than 0.7% and the activation of related genes involved in angiogenesis and glycolysis (6, 7). Under hypoxia, different various molecules and signaling pathways are activated compared with normoxia, including hypoxia-inducible factor-1α (HIF-1α) (8), which mediates cell phenotypic changes. Kong et al. found that serine/threonine kinase (STK33) as a downstream regulator of HIF-1a can regulate the progression of pancreatic cancer, which reveals a part of the PDAC–hypoxia axis (9). Since hypoxia could affect the prognosis of PDAC patients through induction of malignant phenotypes such as invasion and drug resistance (10), discovering more signature genes in the PDAC–hypoxia axis is a necessity. More importantly, taking the microenvironment as a whole may offer new perspectives.

Known as being hypoxic, PDAC is also recognized as an immunosuppressive tumor, which with mutations in immune checkpoints will affect the prognosis (11). Despite the limited success of immune checkpoint inhibitors, such as anti-Programed Death 1 ligand (anti-PD-L1) and anti-cytotoxic T lymphocyte-associated protein 4 (anti-CTLA-4) monoclonal antibodies (12, 13), the mechanism that underlies the complexity of PDAC immune microenvironment deserves to be elucidated. For all we know, the interaction between tumor cells and immune microenvironment components is key to tumor progression and response to immunotherapy (14, 15). Accumulating lines of evidence reveal that hypoxia interacts directly or indirectly with the immune status in the PDAC microenvironment (16, 17), yet the mechanism has been under-investigated. Based on the fact that T-cell infiltration (18), DC function (19), etc. are impaired under hypoxia, Yamasaki et al. suggest in their review that immunotherapy can only be successful if these hypoxia-immune interaction issues are addressed properly (2).

All this evidence adds up to suggest that the interaction between hypoxia and immune status has certain prognostic significance for PDAC. The purpose of this study is to construct the very first hypoxia- and immune-associated prognostic signature model through systematic analysis in hope for its future incorporation into the already existing clinical staging system and an improvement of PDAC prognosis.

## Materials and Methods

### Data Collection and Mining of mRNA Profiles

The messenger RNA (mRNA) expression matrix and the related clinical information were obtained from The Cancer Genome Atlas (TCGA) database (https://portal.gdc.cancer.gov/projects/TCGA-PAAD/) and Genotype-Tissue Expression Project (GTEx) database (https://www.gtexportal.org/), respectively. In this study, specimens with no survival data were eliminated. For further verification, the clinical data and transcriptional information were obtained from PDAC cases in the International Cancer Genome Consortium (ICGC) database (https://dcc.icgc.org/releases/current/Projects/PACA-AU/) and Gene Expression Omnibus (GEO) database (GSE28735, https://www.ncbi.nlm.nih.gov/geo/query/acc.cgi?acc= GSE28735/; GSE62452, https://www.ncbi.nlm.nih.gov/geo/query/acc.cgi?acc= GSE62452/). To maintain the comparability of different databases, FPKM (fragments per kilobase of transcript per million fragments mapped) values of RNA-Seq were log2 transformed. Among them, a total of 364 PDAC samples with complete mRNA expression data and corresponding clinical materials were selected for subsequent analysis.

### Unsupervised Clustering of Hypoxia-Associated Differentially Expressed Genes

“ConsensusClusterPlus” R package, based on the k-means machine learning algorithm, was used to perform an unsupervised consensus clustering, which allows for dividing or condensing cases to multiple different clusters, according to the provided hallmarks or signatures. Besides, hallmark gene sets summarize and represent specific well-defined biological states or processes and display coherent expression. The set of hypoxia hallmark genes (*n* = 200), which is classic and has been used for the hypoxia-associated analysis of other tumors, was acquired based on the Molecular Signatures Database (MSigDB, https://www.gsea-msigdb.org/gsea/msigdb/). In detail, we used the consensus clustering algorithm with 1,000 iterations by sampling 80% of the data in each iteration. The optimal cluster number was confirmed by the Item-Consensus plot, the proportion of ambiguous clustering (PAC) algorithm, and the relative change in the area under the cumulative distribution function (CDF) curves. Two clusters (namely, “hypoxia-low” and “hypoxia-high” groups) were selected for assessing hypoxia status. Kaplan–Meier plots were performed for hypoxia-high and hypoxia-low groups to compare their OS.

### Determination and Annotation of Hypoxia-Associated and Immune-Associated DEGs

By comparing gene transcription profiles of patients from TCGA and GTEx database with R package “limma”, the overall DEGs were identified (|fold change| >2, *p* < 0.05). Pearson correlation was performed to select hypoxia-associated DEGs based on data from overall DEGs and hypoxia hallmark genes with the standard of Cor > 0.8 and *p* < 0.05. On the other hand, we converged overall DEGs and immune hallmark genes as immune-associated DEGs; the latter hallmark genes (*n* = 2,483) were extracted from the immunology Database and Analysis Portal (ImmPort, https://www.immport.org/) database. The potential functions of these hypoxia- and immune-associated DEGs were then ascertained through Gene Ontology (GO) annotation and Kyoto Encyclopedia of Genes and Genomes (KEGG) enrichment Pathway analysis by using the “clusterProfiler” package in R; FDR < 0.05 was considered statistically significant.

### Construction and Verification of Prognostic Signature Associated With Hypoxia and Immune Characteristics

We took the intersection between immune- and hypoxia-associated DEGs, and selected those overlapped genes for univariate Cox regression analysis. Then, they were processed with the Least Absolute Shrinkage and Selection Operator (LASSO) in order to avoid over-fitting and to delete those tightly correlated genes. Tenfold cross-validation was employed to select the minimal penalty term (λ). After that, we established an immune- and hypoxia-associated prognostic signature for the PDAC patients implicating seven hypoxia- and immune-associated DEGs. The formula of the risk score was constructed as follows:


$$Risk\;score=\sum_{i=1}^{n}Coef_{i}*x_{i}$$

where *Coef_i_* represents the coefficients and *X_i_* represents the normalized count of each hub genes. Based on the median risk score, we stratified patients into either a high- or a low-risk group. What is more, the OS and survival-dependent receiver operating characteristic (ROC) curves at 1, 2, and 3 years of prognostic value was performed in TCGA training set and also robustly validated in the GEO and ICGC cohorts.

### Independent Prognostic Value of Signature Genes and Their Relationship With Hypoxia Clusters

Hub genes that formed the prognosis signature were analyzed for their independent prognostic value by univariate Cox regression analysis. Relationship between risk score model and previously constructed hypoxia clusters were analyzed using the R package “pheatmap”. Owing to analyzing the survival conditions of the prognosis signature, the optimal cutoff value was calculated using the R package “survminer”, and the Kaplan–Meier plot of OS of these hub genes was depicted as well.

### Correlations Between Hypoxia-Associated Gene Signature and Clinical Parameters

The subgroup analysis of individual signature genes in the hypoxia- and immune-associated prognostic signature was conducted based on patients’ clinical characteristics. Next, uni- and multivariate Cox regressions were used to verify the prognostic role of the hypoxia- and immune-associated gene signature and select clinical factors. Then, a nomogram was established using R package “rms” based on risk scores and clinical factors with prognostic value (pathological N, primary therapy and age). The predictive effect of the nomogram was validated by assessing the discrimination and calibration plot. To be clear, the calibration curve of the nomogram was plotted to observe the nomogram prediction probabilities against the observed rates.

### Gene Set Enrichment Analysis of the Prognostic Risk Score Model

Gene set enrichment analysis (GSEA) provided by MsigDB was adopted to determine the statistical significance of molecular pathways as well as the consistent heterogeneities between high- and low-risk groups. GSEA software by JAVA program was downloaded from the official website (https://www.broadinstitute.org/gsea/). The gene sets “h.all.v7.4.symbols.gmt” and “c5.go.v7.4.symbols.gmt” were selected as the reference gene set. A pathway with FDR *q* < 0.25 and *p* < 0.05 was defined as statistically significant.

### Relationships of Prognostic Gene Signature With Immunocyte Infiltration

Based on RNA-seq expression matrix of PAAD, CIBERSORT and ESTIMATE algorithms were carried out using R. CIBERSORT algorithm (http://cibersortx.stanford.edu/) was applied in analyzing the differences of immunocyte infiltration status between the high- and low-risk group with regard to 22 immunocyte subunits. Estimation of Stromal and Immune cells in Malignant Tumor tissues using Expression data (ESTIMATE) algorithm was adopted to measure stromal level (stromal score), immunocyte infiltration degree (immune score), and tumor purity in respective PDAC samples as the exploration of risk score model for immune status grouping. Furthermore, the expression status of common immune checkpoints was analyzed between high- and low-risk groups by drawing boxplots.

### Mutation Analysis of the Risk Score Model

The somatic mutation data were acquired from TCGA GDC portal (https://portal.gdc.cancer.gov/). The R package “maftools” was then utilized to draw a waterfall plot to depict the mutation landscape in patients with the high- and low-risk group.

### Statistical Methods

The independent Student’s *t*-test was used to compare the continuous data with normal distribution, and χ^2^ test for categorical data was utilized for pairwise comparisons between subgroups. The Kruskal–Wallis test (one-way ANOVA on ranks) was performed to determine if there are statistically significant differences between multiple groups. The Mann–Whitney *U* test was used to compare differences between two independent groups when the dependent variable is either ordinal or continuous, but not normally distributed. Kaplan–Meier analysis with a log-rank test was used to compare the OS between different subgroups. All statistical analyses were performed using the R programming language (Version 4.0.3). A difference of *p* < 0.05 indicated statistical significance unless specified otherwise.

## Results

### Exploration of Hypoxia-Associated Genes

**Figure 1:**
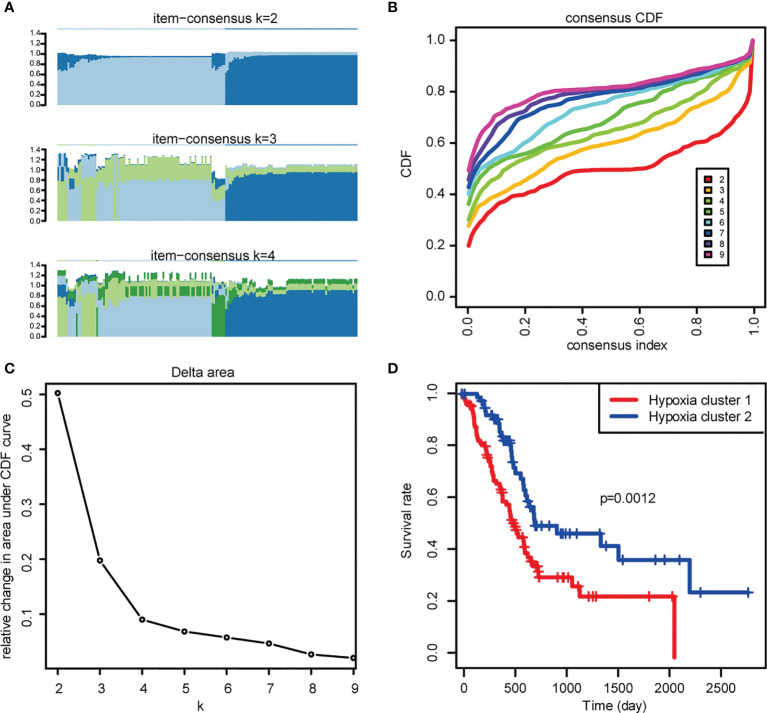
Exploration of hypoxia-associated genes. **(A)** The Item-Consensus Plot represented the chosen optimal cluster number (*k* = 2) for hypoxia genes. **(B)** Consensus values range from 0 to 1. **(C)** The corresponding relative change in area under the cumulative distribution function (CDF) curves when cluster number changes from *k* to *k*+1. The range of *k* changed from 2 to 9, and the optimal *k* = 2. **(D)** Survival curves of patients in Hypoxia cluster-1 and cluster-2.

As the prognosis of PDAC patients with different levels of hypoxia varies, we firstly performed unsupervised clustering analysis to identify distinct hypoxia patterns and stratified patients into two clusters (**Figures 1A–C**). Significant differences were detected across these two clusters upon OS comparison (**Figure 1D**), in which the patients in Hypoxia Cluster 1 (hypoxia-high, with higher extent of hypoxia) has poorer prognosis compared with patients in Hypoxia Cluster 2 (hypoxia-low). This prompted us to continue to explore the relationship between hypoxia levels and prognosis in PDAC patients by looking into hypoxia-associated gene expression.

### Identification and Annotation Hypoxia- and Immune-Associated PDAC DEGs

**Figure 2:**
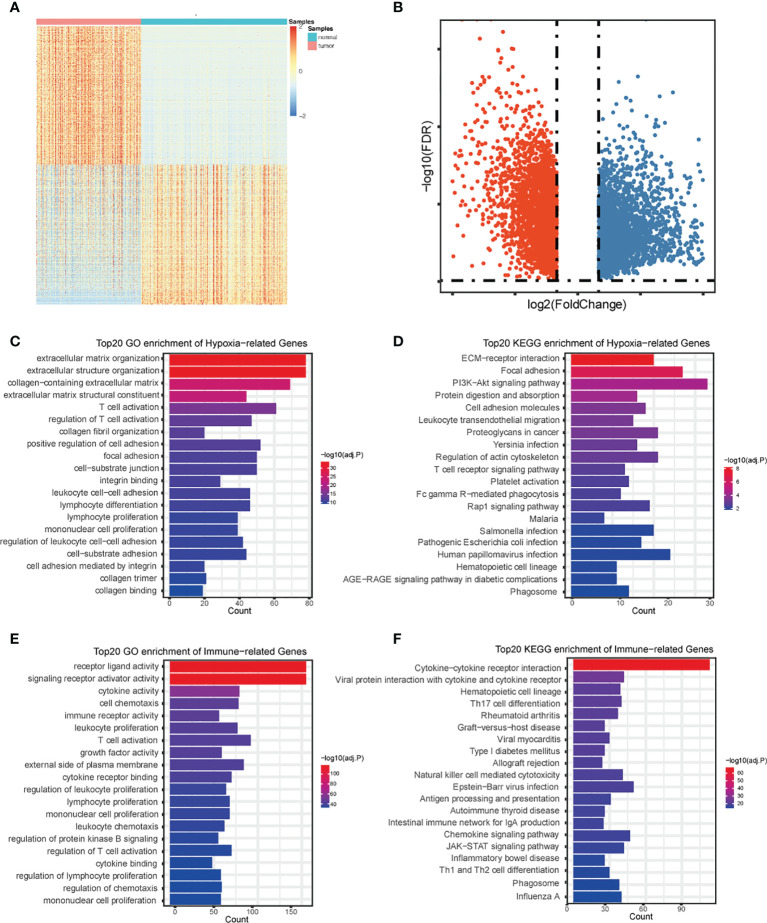
Identification and annotation of DEGs. Heatmap **(A)** and volcano plot **(B)** of differentially expressed genes in PDAC based on data from TCGA and GTEx. **(C)** The top 20 of GO analysis terms of hypoxia-associated DEGs. **(D)** The top 20 most enriched KEGG pathways of hypoxia- associated DEGs. **(E)** The top 20 of GO analysis terms of immune-associated DEGs. **(F)** The top 20 most enriched KEGG pathways of immune-associated DEGs.

By using the R package “limma”, we gathered a total of 5,364 DEGs comparing TCGA with GTEx (**Figures 2A, B**). Further investigation of the relationship between PDAC DEGs and hypoxia marker genes by Pearson correlation analysis showed 682 hypoxia- associated PDAC DEGs. By taking the intersection of PDAC DEGs and 2,483 immune hallmark genes, we identified 528 immune-associated PDAC DEGs. GO and KEGG pathway analysis of DEGs exhibits intriguing results. Some of the most enriched pathways of hypoxic DEGs are immune-associated, including “T cell activation”, “regulation of T cell activation”, “Lymphocyte differentiation”, and “Lymphocyte proliferation” (**Figures 2C–F**), suggesting that the different hypoxic status affecting PDAC prognosis may be related to the activation of immune pathways.

### Development and Validation of Hypoxia- and Immune-Associated Risk Score Model

**Figure 3:**
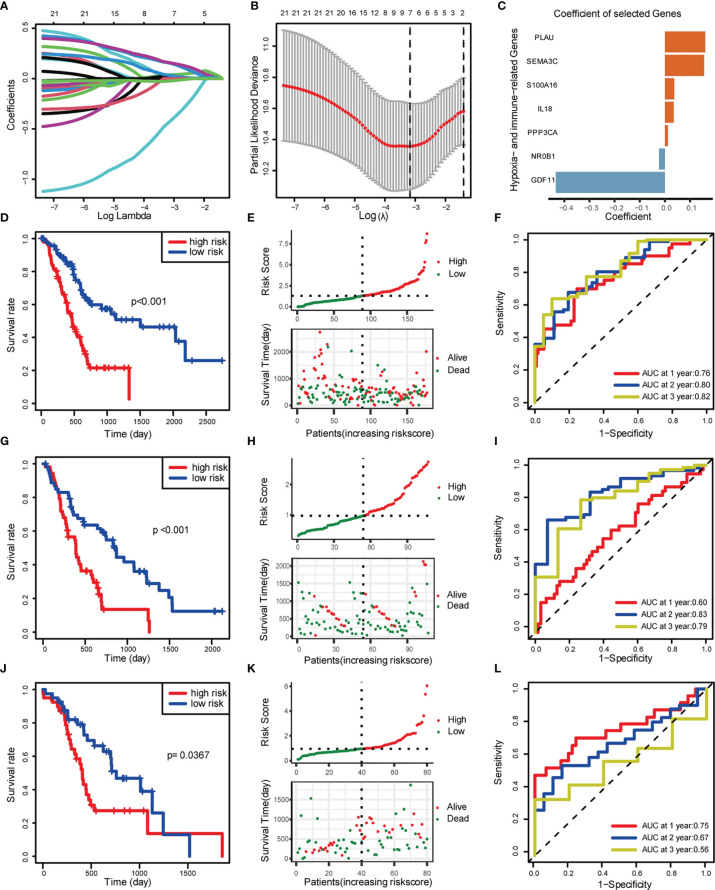
Construction and validation of risk score model. **(A)** LASSO coefficient profiles. **(B)** Selection of the tuning parameter (lambda) in the LASSO model by 10-fold cross-validation based on minimum criteria for OS. **(C)** Coefficient of the seven selected genes. **(D–F)** Construction of TCGA training set. **(D)** OS of TCGA PDAC cohort. **(E)** Distribution of risk score and OS of TCGA training set. **(F)** Survival-dependent ROC curves validation at 1, 2, and 3 years of prognostic value of the prognostic index in TCGA. **(G–I)** Construction of GEO validation set (GSE28735 and GSE62452). **(G)** OS of GEO PDAC cohort. **(H)** Distribution of risk score and OS of GEO. **(I)** Survival-dependent ROC curve validation at 1, 2, and 3 years of prognostic value of the prognostic index in GEO. **(J–L)** Construction of ICGC validation set. **(J)** OS of GEO PDAC cohort. **(K)** Distribution of risk score and OS of GEO. **(L)** Survival-dependent ROC curves validation at 1, 2, and 3 years of prognostic value of the prognostic index in GEO.

These hypoxia-associated DEGs were intersected with the immune-associated DEGs, and altogether 72 overlapping genes were screened for subsequent analysis (**Figure 3A**). By univariate Cox regression analysis, we identified the 22 most relevant DEGs. Afterwards, we chose seven genes for constructing the prognostic signature *via* multivariate Cox regression analysis and LASSO regression, aiming to stratify PDAC patients into two groups with discrete OS, namely, high- or low-risk groups (**Figures 3A–C**). Based on the median risk score, all cases were classified as high- or low-risk group. According to Kaplan-Meier analysis (**Figures 3D, E, G, H, J, K**), high-risk patients had remarkably reduced OS relative to low-risk patients in different sets. Additionally, 1-, 2-, and 3-year OS, based on the values of area under the curve (AUC) for TCGA PDAC cohort, GEO cohort, and ICGC cohort are shown in **Figures 3F, I, L**.

### Independent Prognostic Validation of Seven Signature Genes

**Figure 4:**
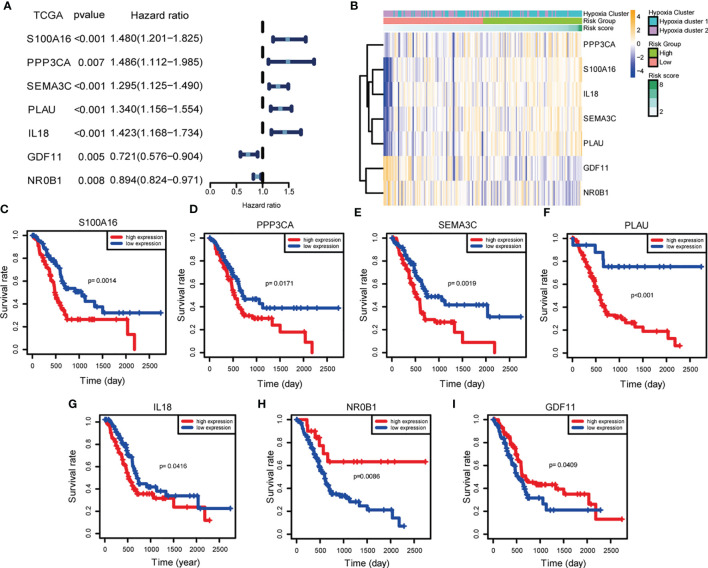
Independent prognostic validation of the seven signature genes. **(A)** Forest plot of univariate Cox regression analysis based on data from TCGA. **(B)** Heatmap of hypoxia- and immune-associated DEGs by unsupervised clustering. The hypoxia cluster, risk group, and risk score as gene annotations were correlated. **(C–I)** Kaplan–Meier survival of each hypoxia- and immune-associated DEGs expression based on data from TCGA.

To gain insight into the independent prognostic value of the seven signature genes in the risk model, we performed univariate Cox regression analysis and found that five of them were harmful to PDAC patients and two of them were beneficial to PDAC patients (**Figure 4A**). On the clustering heat map of the seven genes, we found that the risk model was consistent with the previously established hypoxia clustering, which somehow confirmed our conjecture that hypoxia and immunity may interact in influencing PDAC prognosis (**Figure 4B**). We went on to draw the Kaplan-Meier survival curves to assess the prognostic value of each signature gene, and the results were also consistent with univariate Cox regression analysis (**Figures 4C–I**).

### Correlation of Risk Models With Clinical Characteristics

**Figure 5:**
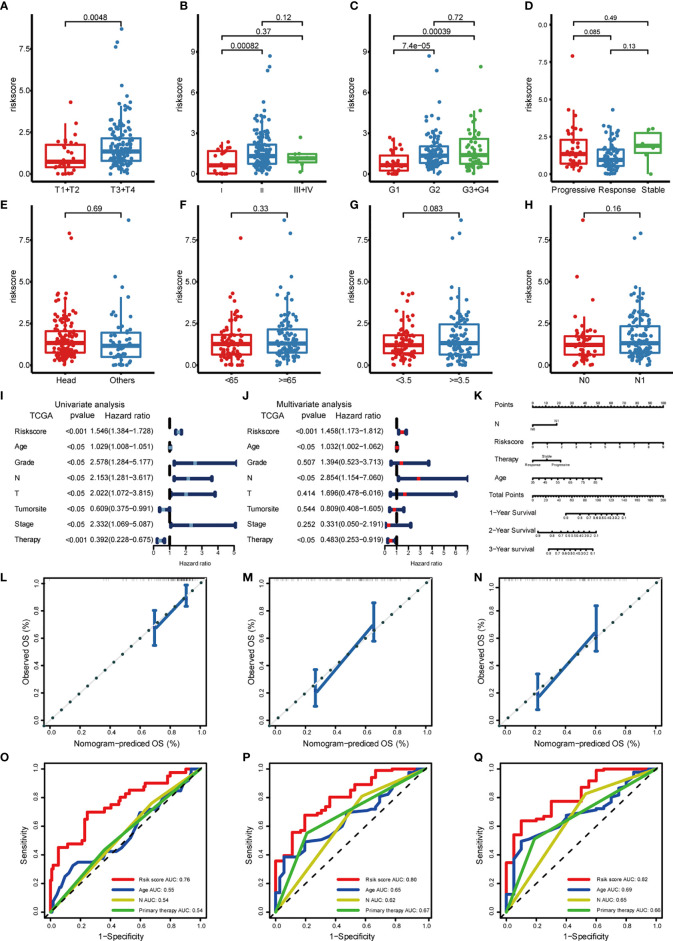
Correlation of risk models with clinical characteristics based on TCGA PDAC cohort. The risk score was significantly correlated with T category **(A)**, stage **(B)**, grade **(C)**, and was not significantly correlated with primary therapy **(D)**, tumor site **(E)**, age **(F)**, tumor size **(G)**, and lymph node invasion **(H)**. **(I)** Univariate survival analysis and **(J)** multivariate survival analysis of clinical characteristics. **(K)** Nomogram predicting OS for PDAC patients. For each patient, four lines are drawn upward to determine the points received from the four predictors. The sum of these points is located on the “Total Points” axis. Then, a line is drawn downward to determine the possibility of 1-, 2-, and 3-year OS of PDAC. **(L–N)** The calibration plot for internal validation of the nomogram. The *y*-axis represents actual survival, and the *x*-axis represents nomogram-predicted survival. **(O–Q)** The time-dependent ROC of the nomogram based on OS.

To investigate whether our risk model correlated with the clinical characteristics of PDAC, we performed the Wilcoxon rank sum test and found that the high-risk group had a more advanced TNM stage and higher tumor grade (**Figures 5A–H**). Considering that the prognostically relevant clinical characteristics differed between the two risk groups, we further investigated whether the risk model had similar or better predictive validity with other PDAC-independent prognostic factors (**Figures 5I, J**). We built a nomogram to predict patients’ OS with three independent prognostic factors including age, primary therapy, N, and the risk score (**Figure 5K**). Calibration plots presented that the nomogram might accurately estimate the mortality (**Figures 5L–N**). The AUCs of the nomogram were 0.76, 0.80, and 0.82 for 1-year, 2-year, and 3-year OS (**Figures 5O–Q**). The above results suggest that the risk model could either work as an independent prognostic factor or be integrated with existing clinical indicators.

### Enrichment Analysis of Hypoxia and Immune Gene Sets in Risk Score Model

**Figure 6:**
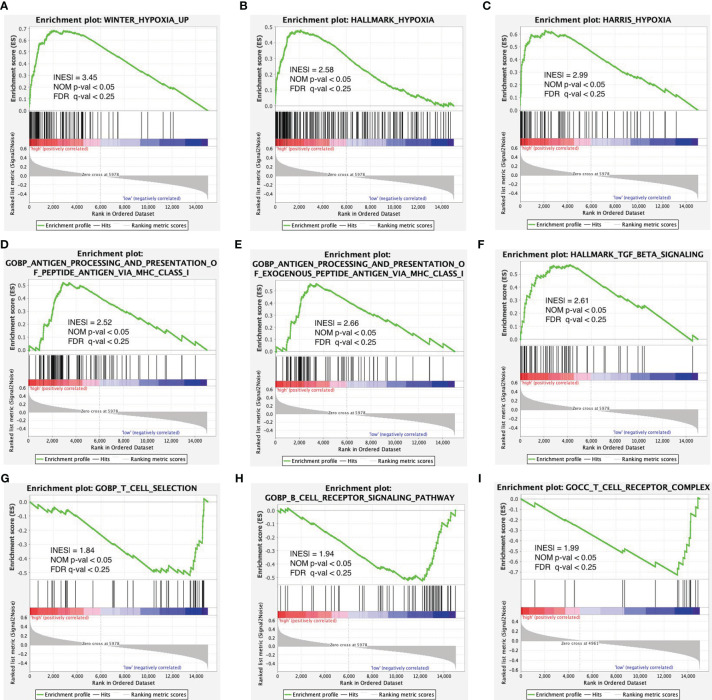
Enrichment plots of hypoxia- and immune-associated gene sets from gene set enrichment analysis (GSEA). GSEA results showing gene sets in **(A)** WINTER_hypoxia_up, **(B)** HALLMARK_hypoxia, and **(C)** HARRIS_hypoxia are differentially enriched in the high-risk group. Enrichment plots of immune-associated gene sets from gene set enrichment analysis (GSEA). GSEA results showing gene sets in **(D–F)** are differentially enriched in the high-risk group while gene sets in **(G–I)** are differentially enriched in the low-risk group.

To further validate the function of the risk model in hypoxia and immunity, we performed GSEA pathway enrichment analysis and found that three hypoxia-associated gene sets were enriched in the high-risk group including WINTER_hypoxia_up, HALLMARK_hypoxia, and HARRIS_hypoxia (**Figures 6A–C**). Of the six immune-associated gene sets, three were enriched in the high-risk group (**Figures 6D–F**) and the other three were enriched in the low-risk group (**Figures 6G–I**). Because the enrichment of the immune-associated gene set is more complex compared with hypoxia-associated gene set, we need further in-depth evaluation of this risk model regarding immune status.

### Relationship Between Risk Score Model and Immune Infiltration

**Figure 7:**
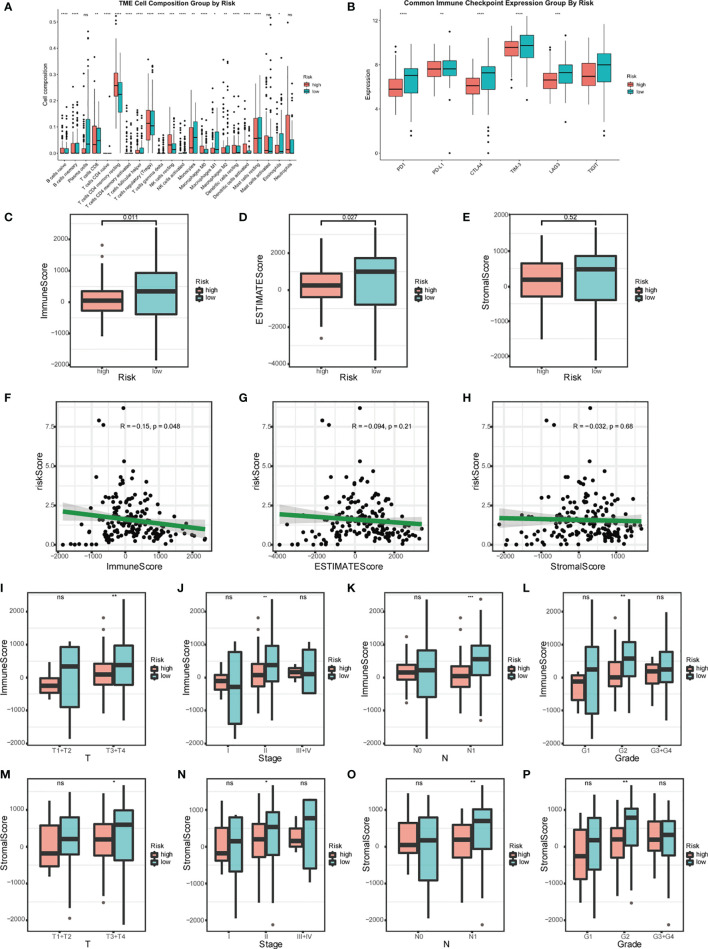
Relationship between risk model and immune status. **(A)** Analysis of the immunocyte infiltration degrees in both groups regarding 22 immunocyte subunits. **(B)** Boxplots visualizing different immune checkpoint expression between high-risk and low-risk patients. **(C–E)** Estimation of risk score based on TCGA. The relationship between the risk signature and Immune Score, ESTIMATE Score, and Stromal Score. **(F–H)** Scatter plot of Immune Score, ESTIMATE Score, and Stromal Score. Analysis of different immune status **(I–L)** and stromal status **(M–P)** in high- and low-risk groups of TCGA PDAC cohort and its correlation with clinical features. (*p < 0.05; **p < 0.01; ***p < 0.001; ****p < 0.0001; ns, p > 0.05).

High-risk patients showed higher immunocyte infiltration degrees of naïve B cells, CD4 memory resting T cells, regulatory T cells (Tregs), resting NK cells, M0 macrophages, resting dendritic cells, and activated dendritic cells, while the low-risk group showed higher infiltration degrees of memory B cells, CD8 T cells, follicular helper T cells, monocytes, M1 macrophages, M2 macrophages, resting mast cells, and eosinophils (**Figure 7A**). The ESTIMATE score showed that the high-risk group had lower stromal and immune scores compared to the low-risk group (**Figures 7C–H**). In clinically subgroup analysis, immune scores and stromal scores were significantly lower in the high-risk group than in the low-risk group in T3T4, Stage2, N1, and Grade2 (**Figures 7I–P**). The immune checkpoint expression levels were also significantly lower in the high-risk group than in the low-risk group (**Figure 7B**). Combining these results, we found that different risk groups of pancreatic cancer can accurately suggest the level of immunity, and the overall level of immune response was lower in patients in the high-risk group than in the low-risk group.

### Relationship Between Risk Score Models and PDAC Mutations

**Figure 8:**
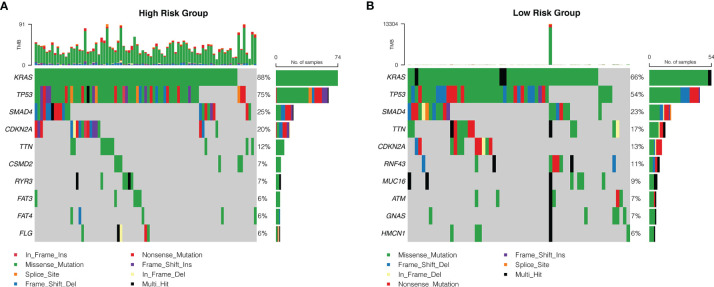
The mutation frequency of genes in patients with PDAC from TCGA database. Correlation between the high-risk group **(A)** and the low-risk group **(B)** with mutations is presented. Each column represented individual patients. The upper barplot showed TMB. The number on the right indicated the mutation frequency in each regulator. The right barplot showed the proportion of each variant type.

An oncoplot showed the most frequently mutated PDAC genes in the high-risk and low-risk groups (**Figures 8A, B**). The mutation burden (TMB) is significantly higher in the high-risk groups. What is more, four PDAC mutated genes (*KRAS*, *TP53*, *SMAD4*, and *TTN*) were more frequently mutated and had a richer mutation spectrum in the high-risk groups. The other six PDAC mutated genes (*CDKN2A*, *RNF43*, *MUC16*, *ATM*, *GNAS*, and *HMCN1*) were less frequently mutated in the high-risk group than in the low-risk group, and had a narrow mutation spectrum.

## Discussion

The PDAC microenvironment contains various factors including hypoxia, immune cell infiltration, and fibrosis (20). Hypoxia enhances PDAC proliferation, metastasis, and resistance to radiotherapy and chemotherapy (21). Meanwhile, the immune microenvironment of PDAC also affects tumor progression (22). Considering the development of a prognostic strategy, targeting a single factor may be insufficient to classify PDAC patients, and we discussed in this study the possibility that PDAC hypoxia and immune microenvironment together will elucidate prognosis of PDAC.

Prognosis was worse in our highly hypoxic cluster (Hypoxia Cluster 1), which is in line with a similar theory of Chiou et al, who claims BLMP1, induced by hypoxic microenvironment, can damage prognosis of PDAC by promoting metastasis through regulating hypoxia-associated gene expression (23). Liu et al. found that the anti-cancer factor CF129 is poorly expressed in the hypoxic microenvironment and thus fails to ubiquitinate p53 protein, which in turn leads to worse prognosis (24).

Due to the complexity of hypoxia and immune activity within the tumor microenvironment (TME) (25), we conducted GO and KEGG pathway analysis for hypoxia-associated DEGs and discovered that several immunoregulatory pathways were enriched. Among them, T-cell activation was hampered by hypoxia-induced myeloid-derived suppressor cells (MDSC) in colorectal cancer (26). Lymphocytes were affected by tumor-derived exosomes in the context of hypoxia, which subsequently regulates MDSC function in a miR-21/PTEN/PD-L1 axis in oral squamous cell carcinoma (27). Importantly, there are also hypoxia-associated pathways enriched in immune DEGs. ECM transcriptional program dysregulation is correlated with the activation of TGF-β signal in cancer-associated fibroblasts and is linked to immunosuppression in immunologically active tumors (28). Together with these researches, our result linked hypoxia with immunity.

With LASSO analysis, we identified seven signature genes (S100A16, PPP3CA, SEMA3C, PLAU, IL18, GDF11, and NR0B1). Fang et al. revealed that S100A16 promotes PDAC progression through FGF19-mediated AKT and ERK1/2 signaling (29). Li et al. demonstrated that S100A16 induces the EMT to promote the metastasis of PDAC, which is mediated by TWIST1 and STAT3 signal (30). Zhuang et al. found that overexpression of S100A16 was significantly associated with a higher T stage, advanced histologic grade, and worse prognosis, and may impair the infiltration and cytolytic activity of CD8+ T cells through focal adhesion-Ras-stimulating signal pathway (31). As for IL-18 being a double-edged sword, it alone promotes carcinogenesis, but when combined with NF-κb inhibitor, it exhibits an anti-tumor effect (32). Sun et al. believed that the feedback loop of NF-κb signal and its downstream IL-18 is the key to understanding PDAC metastasis (33). Xu et al. demonstrated that overexpression of SEMA3C is correlated with poor prognosis of PDAC patients by activating ERK1/2 signaling pathway (34). For PPP3CA, PLAU together with the other two “nice” genes identified by us, mechanistic studies of good quality are rare, which provide underlying targets for experimental design to uncover molecular mechanisms. Importantly, the risk score model is correlated with previously established hypoxia clusters, which strengthened the link between hypoxia and immunity. The nomogram incorporating the seven-gene signature and clinicopathological parameters showed great prognostic potency, which may enable clinicians to determine an individual patient’s prognosis. In other studies of this field, Yan et al. identified a four-gene signature (LYRM1, KNTC1, IGF2BP2, and CDC6) significantly associated with progression and prognosis of pancreatic cancer (35). More recently, Feng et al. also discovered a seven-gene signature (ASPH, DDX10, NR0B2, BLOC1S3, FAM83A, SLAMF6, and PPM1H) for prognosis prediction of PDAC patients (36). No overlap was identified between the seven-gene prognostic signature we developed and those previously defined. Besides, the methodology of signature construction we adopt is a more unsupervised and unbiased way. To our knowledge, this is the first prognosis signature risk score model ever built containing both key factors of PDAC microenvironment, hypoxia, and immunity. Taken together, our prognostic signature was identified to be superior or comparable to the previous defined signatures.

By GSEA, we discovered that all four hypoxic gene sets were enriched in the high-risk group, confirming that pancreatic cancer is a hypoxic tumor (37). Interestingly, half of immune genes were enriched in the high-risk group. To be specific, Cave et al. reported that inactivation of TGF-β1-Smad2/3 signaling in PSCs strongly reduced the aggressiveness of PDAC cells by rescuing L1 cell adhesion molecule (L1CAM) (38). Yamamoto et al. reported that in PDAC, major histocompatibility complex class I (MHC-I) molecules are selectively targeted for lysosomal degradation by an autophagy-dependent mechanism (39). The other half of immune gene sets were enriched in the low-risk group. Akce et al. summarized characteristics of chimeric antigen receptor (CAR) T-cell therapy, which utilizes genetically engineered T cells that are redirected to specific cancer-associated antigens to elicit potent cytotoxic activity (40). Burger et al. summarized that B cells and BCR-related kinases, such as BTK, play a role in the microenvironment of PDAC, which could be targeted to achieve great anticancer activity (41). All of the mentioned gene sets regardless of their enrichment status are contributing to our promising prognostic risk score model.

The different expression profile from CIBERTSORT in our risk score model is highly consistent with current studies on immune cell infiltration. Gunderson et al. discovered that in the mature tertiary lymphoid structure-positive group, a sign of misery prognosis, patients have higher expression of naïve B cell (42). Ma et al. demonstrated that the combination of anti-PD-1 inhibitory and anti-OX40 agonist antibodies reduces the proportion of regulatory T cell in PDAC (43). Induced pluripotent stem cell-based cancer vaccine could also reduce immunosuppressive CD4+ T regulatory cells (44). Notable is the extinct higher proportion of resting memory CD4+ T in our high-risk group, which is consistent with the finding of resting central memory CD4+ T cells that predicted a worse prognosis from Gu et al. (45). Spear et al. demonstrated in a murine study that B-cell memory infiltration is an immunostimulatory factor that might support the adaptive antitumor immune response (46), which is consistent with its high expression in our low-risk group. Taken together, our risk score was correlated with the immunosuppressive microenvironment of the tumor.

In the ESTIMATE analysis, we found that both the immune score and the stromal score were higher in the lower-risk group, which is easy to comprehend because of the consensus that PDAC is an immunosuppressive tumor with low immunogenicity while being extra malignant (47). To be specific, a tumor with lower immune score is correlated with higher risk score in T3, T4, and Stage II PDAC with regional lymph node metastasis, which is consistent with the finding of Yamasaki et al. that larger tumors are more likely to develop hypoxia and metastasis through hypoxia-related pathways (2). The stroma scores are high in both groups. According to Gorchs et al., carcinoma-associated pancreatic fibroblasts (CAFs) co-inhibit effector CD4+ and CD8+ T cells to damage immunity (48). Our risk score model is able to accurately stratify patients according to their immune microenvironment.

Immune checkpoint assays showed lower levels of immune checkpoint expression in the high-risk group (49). Considering that current immunotherapy against immune checkpoints in pancreatic cancer constantly fails to achieve satisfactory efficacy, we believe that this may be related to the low level of immune checkpoint expression in high-risk patients (47). Interestingly, though not significant, the stroma score in the low-risk group is higher, which, according to Gorchs et al., is because CAFs could induce the expression of immune checkpoints on CD4+ and CD8+ T cells (48). The silver lining is, for the low-risk score patients, who have high immune checkpoint expression, they could benefit from immunotherapy. What is more, there’s a synergistic effect when a combinatorial approach of immunotherapy in conjunction with other modalities is being exploited (50). In this case, we believe combining hypoxia and immunity not only serves as a prognostic classifier but could guide treatment.

The limitations of our work are as follows: Firstly, since all information and tissues were obtained retrospectively from public databases, the two independent external validations we performed cannot cover all variations of PDAC cases from all relevant regions. Secondly, since the number of TCGA pancreatic cancer cases is not large enough, some statistical differences were not ideally significant. Thirdly, since the external and internal part of a tumor differs in microenvironmental characteristics, taking the tumor as a whole may not be able to differentiate the hypoxic and immune status of different tumor sites. For possible differences within and outside the tumor, the use of single-cell RNA sequencing combined with spatial transcriptomic analysis can be considered to potentially address this issue. For the seriousness of scientific research and the novelty of risk score model, we would love to see our results going through more thorough validation in well-designed multicenter prospective studies.

## Data Availability Statement

The original contributions presented in the study are included in the article/supplementary material. Further inquiries can be directed to the corresponding authors.

## Author Contributions

XY and HZ designed the study. WG and DC searched the data from database. DC, WG, HH, and LZ performed analysis of the data. HH and DC wrote the manuscript. HZ and XY revised the manuscript. WG modified the language. All authors contributed to the article and approved the submitted version.

## Funding

This research was funded by the National Natural Science Foundation of China (No. 81873589); the National Natural Science Foundation For Young Scientists of China (No. 82000614); the National Natural Science Foundation for Young Scientists of Hunan Province, China (2020JJ5876); and the Science and Technology Project of Changsha, Hunan, China (No. kq2004146).

## Conflict of Interest

The authors declare that the research was conducted in the absence of any commercial or financial relationships that could be construed as a potential conflict of interest.

## Publisher’s Note

All claims expressed in this article are solely those of the authors and do not necessarily represent those of their affiliated organizations, or those of the publisher, the editors and the reviewers. Any product that may be evaluated in this article, or claim that may be made by its manufacturer, is not guaranteed or endorsed by the publisher.
